# Phase-Coherent Transport in Two-Dimensional Tellurium Flakes

**DOI:** 10.1021/acsaelm.5c01853

**Published:** 2026-02-04

**Authors:** Mohammad Hafijur Rahaman, Nathan Tanner Sawyers, Mourad Benamara, Trudie Culverhouse, Gokul Acharya, Durga Venkata Maheswar Repaka, Qiyuan He, Hugh O. H. Churchill, Dharmraj Kotekar-Patil

**Affiliations:** † Department of Physics, 3341 University of Arkansas, Fayetteville, Arkansas 72701, United States; ‡ Institute for Nano Science and Engineering, University of Arkansas, Fayetteville, Arkansas 72701, United States; § Institute of Materials Research and Engineering, 208718 Agency for Science Technology and Research, (A*STAR) Singapore 138634, Republic of Singapore; ∥ Department of Chemistry, School of Natural Sciences, University of Manchester, Manchester M13 9PL, United Kingdom; ⊥ Department of Materials Science and Engineering, 121844 City University of Hong Kong, 83 Tat Chee Avenue, Kowloon, Hong Kong 999077, China; # MonArk NSF Quantum Foundry, University of Arkansas, Fayetteville, Arkansas 72701 United States

**Keywords:** fabry−perot, quantum dot, spin splitting, phase coherent, chiral material, tellurium, semiconductor, weyl physics

## Abstract

Elemental tellurium (Te) is a compelling van der Waals material due to its interesting chiral crystal structure and predicted topological properties. Here, we report the fabrication and comprehensive quantum transport study of devices based on Te flakes with varying thicknesses. We demonstrate a hole mobility reaching up to 1000 cm^2^/V·s in a 17 nm thick flake at 30 K. At deep cryogenic temperatures (<50mK), the transport characteristics transition from Coulomb blockade in the low carrier density regime to pronounced Fabry-Pérot (F–P) interference at higher densities. Notably, the visibility of these F–P oscillations is significantly enhanced in the thinner flake device. The application of a magnetic field reveals a clear Zeeman splitting of the conductance peaks. The rich variety of quantum transport phenomena (Coulomb blockade, F–P interference, Zeeman splitting) observed underscores the high quality of our thin Te flakes and establishes them as a promising material for exploring physics and device concepts, such as topological superconductivity and low-power spintronic applications.

## Graphical abstract

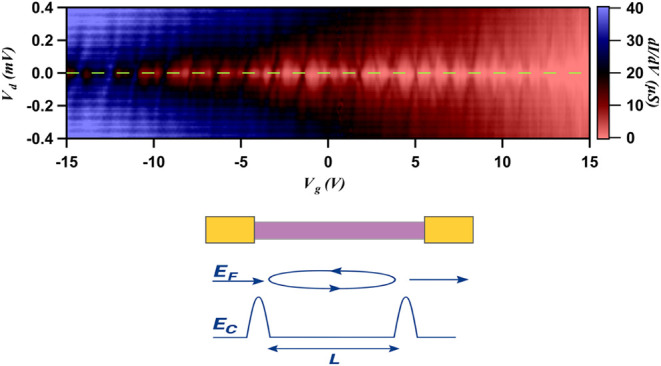

## Introduction

The exploration of quantum phenomena in two-dimensional (2D) van der Waals materials has been a central and highly active theme in modern condensed matter physics. Seminal discoveries in materials like graphene^1^ and transition metal dichalcogenides^2^ have not only revealed a host of new physical effects^3–6^ but have also opened pathways for next-generation electronic and optoelectronic devices.^7–13^ A key frontier in this field is the search for and characterization of materials with nontrivial electronic topology, which promise to host exotic quasiparticles and protected quantum states. This pursuit has driven research beyond established 2D systems (e.g., graphene and TMDCs) toward elemental materials whose unique crystal structures and electronic properties may offer new platforms for investigating fundamental physics and realizing new device functionalities.

Within this landscape, elemental tellurium (Te) has recently emerged as a uniquely compelling candidate. Its chiral, one-dimensional chain-like crystal structure^14,15^ (Figure 1a) gives rise to theoretically predicted Weyl-semimetal characteristics and the potential for nontrivial topological electronic states.^16,17^ While the properties of bulk Te are well-documented,^18–21^ realizing its full potential for quantum device applications requires the fabrication of high quality, thin crystalline flakes where quantum phenomena are enhanced and can be systematically investigated. While mechanical exfoliation and chemical vapor deposition (CVD) are standard for producing 2D films from van der Waals materials, creating large-area thin 2D tellurium has been challenging. Previous attempts to grow tellurium nanostructures resulted in either quasi-1D forms or relatively small 2D nanostructures, unsuitable for transport studies.^22–24^ A recently introduced liquid-based synthesis method, however, successfully produces large-scale 2D tellurene films.^25,26^ With dimensions exceeding tens of micrometers and variable thicknesses, these high-quality nanofilms have been validated and present a promising new platform for investigating both electronic devices and magneto-transport properties. The electronic quality reported for Te grown using the hydrothermal method is comparable to that obtained from other growth techniques, such as chemical vapor deposition,^27,28^ as evidenced by the measured mobilities. This level of performance is maintained despite the solvent-based isolation of the flakes, indicating that the hydrothermal growth route can produce Te with an electronic quality comparable to other established synthesis methods.

**Figure 1:**
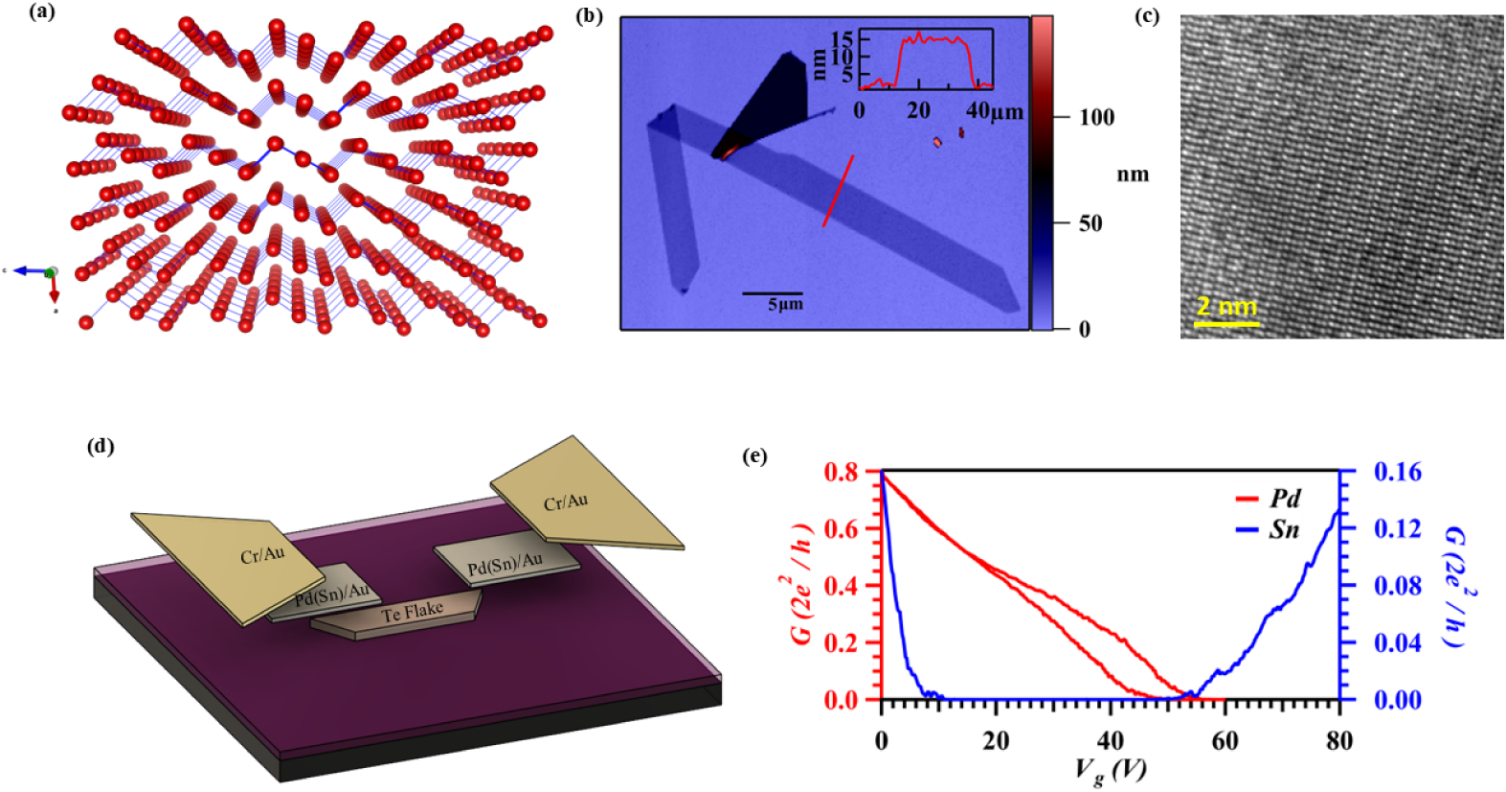
(a) Schematic of the Te crystal exhibiting helical Te chains. (b) Atomic force microscope micrograph of a Te flake of thickness 13 nm. The inset shows the linecut of the flake height. Scale bar in the inset is 5 μm. (c) Transmission electron micrograph of the Te flake showing its atomic structure. (d) Schematic of the device structure, including Pd (Sn)/Au contacts on a silicon substrate. (e) Transconductance trace in Sn/Au and Pd/Au contacted Te devices.

The recent discovery of emergent topological phenomena in elemental tellurium, such as Weyl nodes and signatures of the quantum Hall effect, has sparked significant interest in leveraging its chiral structure to explore nontrivial electronic properties.^29–32^ The intersection of bulk Weyl bands with certain crystal surfaces leads to the emergence of surface Fermi arcs, which serve as a hallmark of Weyl fermions. An especially intriguing manifestation of this topology is the formation of Weyl orbitals, in which charge carriers follow a closed trajectory that links opposite surfaces, originating from a Fermi arc on one surface, propagating through bulk Weyl states, and reemerging on the other side. Observation of such orbits necessitates phase-coherent transport between the two surfaces, a challenging condition for bulk crystals, but one that can be realized in thin Te flakes.

Here, we report on the successful fabrication and comprehensive low-temperature transport study of field-effect devices based on Te flakes. We observe a rich spectrum of quantum transport features, including a clear observation of a Coulomb blockade and pronounced Fabry-Pérot interference (F–P). F–P interference is often observed in highly crystalline materials (e.g., graphene, group III–V and IV semiconductors, etc.)^33–37^ reflecting high crystal quality of our thin Te flakes and its suitability for quantum transport studies. The length scales estimated in these studies, e.g., the mean free path and phase coherence length, provide a measure of the spatial range over which key topological phenomena can be probed. For instance, the detection of Weyl orbitals requires a phase-coherent transport regime, offering valuable design considerations for future studies on thickness-dependent topological properties in 2D Te.

## Experimental Results

### Crystal Characterization and Device Fabrication

Tellurene (Te) flakes were synthesized using a hydrothermal method^26^ and transferred onto heavily doped p-type silicon substrates capped with a 285 nm SiO_2_ layer, which served as a global back gate to modulate carrier density in the Te channel. The as-grown Te flakes typically display a trapezoidal geometry (Figure 1b) with the long edge aligned along the helical axis of the crystal, aiding the alignment with the device orientation. Structural characterization of 2D tellurium flakes is performed using transmission electron microscopy. Figure 1c shows a high-angle annular dark-field scanning transmission electron microscopy (HAADF-STEM) image. The resolved atomic structure shows helical tellurium chains with 3-fold screw symmetry along the [0001] direction. Measured interplanar spacings of 2.3 and 6.0 Å correspond to the (1̅210) and (0001) lattice planes of 2D tellurium, respectively.

Two-terminal field-effect transistor devices were defined via maskless photolithography, and metal contacts, either tin (Sn) or palladium (Pd), were deposited using electron beam evaporation. A schematic of the different device layers is shown in Figure 1d. All devices in this study employed Te flakes with thicknesses ranging from ∼13 to 60 nm. The device measurements presented in the main text of this manuscript display device measurements performed on flakes with thicknesses of 17 and 16 nm, while measurements on other thicknesses are presented in Supporting Information (Supporting Figure S1). Sn/Au-contacted devices exhibit ambipolar transport as shown in Figure 1e.

Devices fabricated using thinner flakes (13–20 nm) exhibited a strong gate tunability with on/off ratios exceeding 10^3^ (limited by the off-state current in the measurement setup) (Figure 2a), while thicker flakes showed a weaker gate response with on/off ratios of ∼10 (Supporting Figure S2). This may be affected by the electric field penetrating the flakes that change the carrier density in the Te flakes. In thinner flakes, we expect to have a strong electrostatic gate effect compared to thicker flakes as the electric field is screened in thicker flakes, resulting in a weaker gate effect. Moreover, in thinner flakes, the bandgap can increase due to enhanced quantum confinement, which in turn may lead to a reduced off-state current relative to thicker flakes.

**Figure 2:**
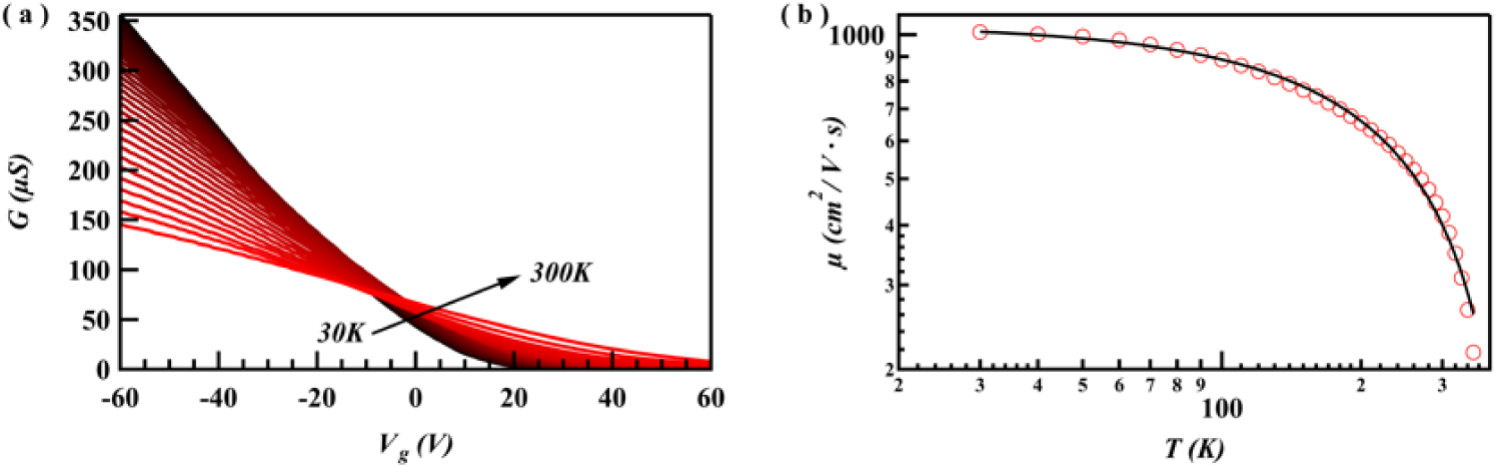
Temperature-dependent electrical transport properties of the device. (a) 2D map of device conductance vs temperature and applied backgate voltage. (b) The extracted field-effect mobility as a function of temperature. At higher temperatures (rapid drop in mobility above *T* > 100 K), the mobility is limited by phonon scattering, as evidenced by its power law T^–γ^. Conversely, the mobility saturates at a high value at low temperatures, indicating that scattering from charged impurities is not a dominant factor in this device.

### Temperature-Dependent Mobility

We measure these devices under “high” and “low” bias conditions, each probing a different transport regime. In the high-bias regime, many states participate in transport, washing out any individual/few level transport effects. In this regime, we measure conductance (*G*) by applying a relatively large source-drain voltage (*V* = 0.1 V) as a function of backgate voltage (*V*_*bg*_) at various temperatures and extract field-effect mobility. Figure 2a displays a transconductance trace in a 12 nm thick Te flake device (device dimensions with width *W* = 5.8 μm and channel length *L* = 11.5 μm). By sweeping *V*_bg_ from −60 to 60 V, *G* drops and pinches off at ∼5 V consistent with holes as the dominant charge carriers, exhibiting p-type behavior.^25,38^ From the linear region of the transconductance trace, field-effect mobility can be extracted using the equation:

$$\mu =\frac{L}{C_{ox}\times W}\frac{dG}{dV_{bg}}$$

where *C*_ox_ is the gate capacitance.

The field-effect mobility, calculated using the equation above, is depicted as a function of temperature in Figure 2b. By fitting the temperature dependence of the mobility, one can gain insight into the dominant scattering mechanisms. We fit the mobility using the general expression:

$$\mu \left(T\right)=\mu_{0}+AT^{\gamma }$$

where *μ_0_* is the saturation mobility, *A*is a fitting constant, and *γ* is the power-law exponent. The black trace in Figure 2b represents the fit to the measured mobility, with *μ_0_* and *γ* treated as free parameters. In the phonon-limited transport regime, charge carrier mobility is expected to decrease with increasing temperature according to the power law relation μ ∼ T^–γ^. The value of the exponent, γ, distinguishes between different scattering mechanisms; acoustic phonon scattering typically results in γ = 1–1.5. Our analysis yielded a γ value of approximately 1.26, indicating that acoustic phonons are the predominant scattering source limiting mobility in our tellurium device. This finding is consistent with similar observations in other two-dimensional material systems, such as MoS_2_.^39,40^ Additionally, the observed mobility saturation at low temperatures strongly suggests minimal influence from charge impurity scattering (up to 30 K).^41^ Mobility measured in another device is shown in Supporting Figure S3, which is comparable to the previously reported values with similar Te thickness.^42^ The mobility values reported here are field-effect mobilities, which generally serve as lower bounds relative to Hall mobility measurements.

In our Te nanoflake devices, a clear metal–insulator transition (MIT) is observed (Figure 2a), where the current decreases with increasing temperature at negative gate voltages (−7 V and below metallic behavior) and shows conventional semiconducting temperature dependence at more positive gate voltages. This MIT can be understood within a classical percolation framework,^43^ where insufficient screening in 2D Te at low carrier density leads to an inhomogeneous potential landscape and the formation of insulating puddles. At higher carrier densities, enhanced screening restores metallic transport. Importantly, the position of this MIT boundary is thickness dependent: in thinner flakes, stronger quantum confinement and weaker screening allow tuning into the insulating regime with gate voltage more easily, whereas in thicker flakes (Supporting Figure S3a), improved screening, narrow bandgap and more bulk-like character suppress gate tunability, resulting in predominantly metallic transport and a diminished MIT signature.

Sn-contacted devices exhibit an ambipolar transport nature, due to the narrow band gap in Te and a more left-shifted threshold voltage for valence band transport (Figure 1e). Conductance band transport appears at higher positive gate voltages. From the slopes of the transconductance characteristics, hole mobility was found to exceed electron mobility, possibly due to higher contact resistance for electrons arising from Schottky barriers at the conduction band compared to valence band.

### Quantum Transport Regime

In the low-bias regime, the transport window narrows, restricting charge transport to a limited number of energy states. This selective probing enables the observation of fine quantum features that would otherwise be obscured at higher biases. At these low energies, comparable to or smaller than the sub-band spacings, quantum phenomena such as Coulomb blockade and interference effects become prominent.

Figure 3a presents the two-terminal conductance *G* as a function of *V*_*bg*_ measured at a base temperature in a dilution refrigerator (<50 mK) in a device using Te thickness of 16 nm. The device channel length is 18 μm, and W is 6 μm. Strong oscillations in *G* are evident, indicating resonant features in the Te channel density of states. Two distinct features are observed. Figure 3b exhibits a schematic of the mechanism in the device that gives rise to these two features. To investigate these features further, we performed bias spectroscopy, mapping *G* as a function of source-drain bias *V* and *V*_*bg*_, as shown in Figure 3c. First, we observe diamond-shaped conductance domains visible near the pinch-off (low carrier density region), a characteristic of Coulomb blockade, as shown in Figure 3d. These “Coulomb diamonds” arise from single-hole tunneling through a confined region weakly coupled to the leads (low barrier transparency). In this limit, hole transport is governed by Coulomb repulsion, enforcing sequential tunneling and resulting in sharply defined energy levels due to the increased lifetime of charge carriers (Figure 3, bottom schematic).

**Figure 3:**
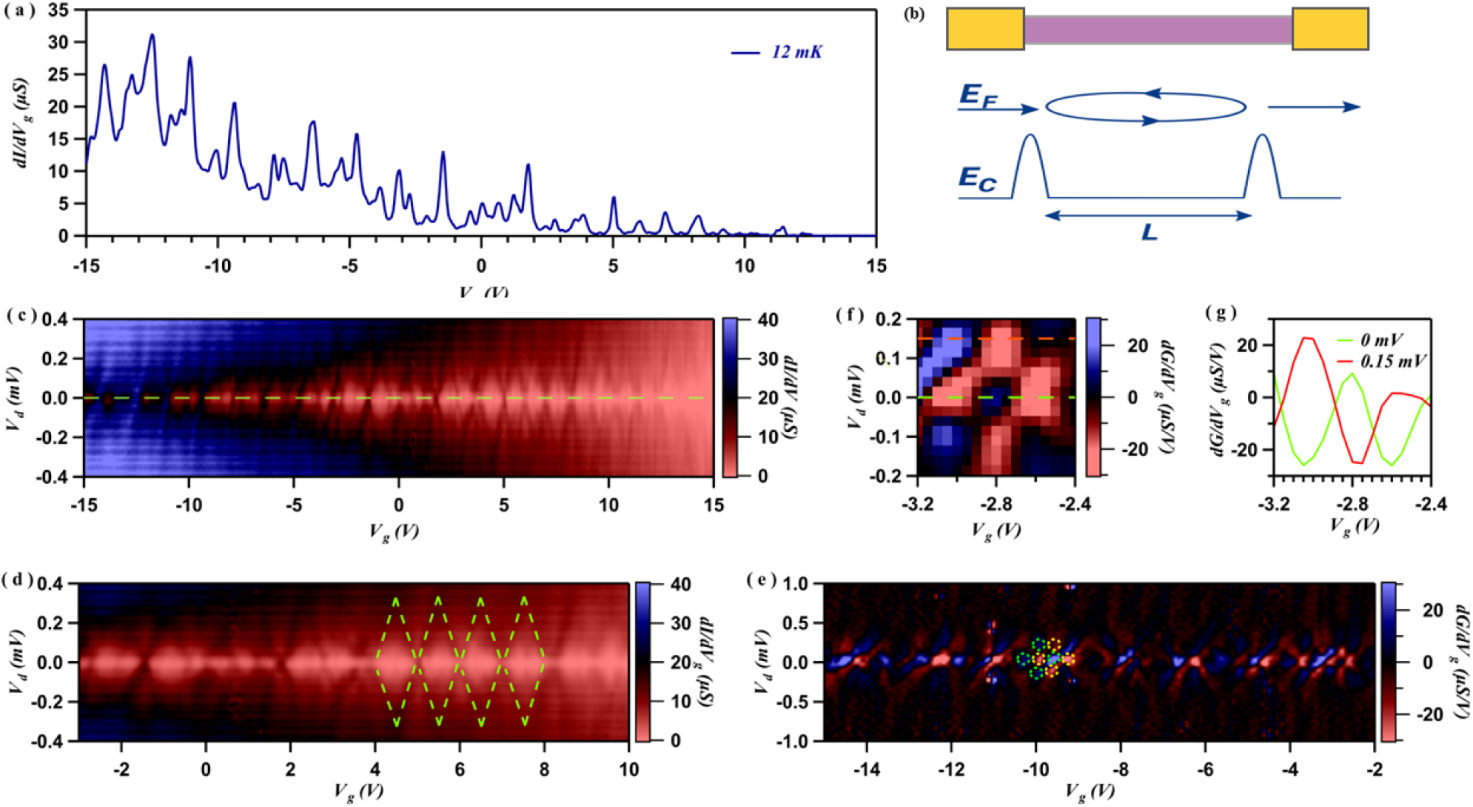
Coulomb blockade and Fabry–Pérot interference in a hole gas. (a) Differential conductance measured in a thin Te device at a base temperature of 12 mK, displaying strong oscillations as the backgate voltage is swept. (b) A schematic illustrating the origin of the two distinct oscillatory phenomena observed. (c) A stability diagram plotting differential conductance as a function of source–drain bias and backgate voltage. The plot reveals two transport regimes: Coulomb blockade, characterized by Coulomb diamonds, dominates at low carrier density, while Fabry-Pérot interference emerges at higher carrier densities. (d, e) Magnified views of the regions in (c) dominated by Coulomb blockade (marked with green dashed lines) and Fabry–Pérot interference (green and yellow dashed circles), respectively. (f) A further zoomed-in view of the Fabry–Pérot regime, highlighting the checkerboard conductance pattern. (g) A linecut from panel (f) demonstrates a phase shift of π in the conductance oscillations, a signature of alternating constructive and destructive interference.

From the size of the Coulomb diamonds, we estimate a charging energy of approximately 400 μeV, corresponding to a self-capacitance of ∼400 aF. From the capacitance, we estimate the QD size to be ∼ 700 nm, fraction of the device dimension, suggesting the QD is formed within a fractional segment of the flake originating from potential fluctuations in the Te flake in the low carrier density region. At low gate voltages, charge carriers tend to accumulate near the Te/SiO_2_ interface. The resulting potential fluctuations in the channel may originate from an inhomogeneous charge distribution at low carrier densities or from surface roughness induced by the underlying SiO_2_ substrate, similar to that observed in other TMDCs, as well as defects introduced during flake isolation. These surface imperfections are closely related to the microscopic details of the liquid exfoliation process.

In the more negative *V*_bg_, the second feature, where *G* pattern displays a checkerboard pattern (second derivative, Figure 3e) signifies an F–P interference regime,^35^ where more transparent barriers are formed, and the discrete charge state (in case of Coulomb blockade) is no longer well-defined. In this open-dot regime, hole wave functions interfere constructively and destructively between two partially reflective barriers, forming a quantum cavity. This is more evident in the more negative *V*_bg_ range (Figure 3b central schematic).

Systematically, the position of the *G* peaks shifts linearly upon increasing *V*, as expected for Fabry–Pérot interference.^44,45^ The shift is also illustrated in Figure 3(f) (displaying a phase shift of π due to constructive and destructive interference). This is clearly visible in a line cut shown in Figure 3g for measurements taken at *V* = 0 and 0.15 mV. From Figure 3(g), the bias needed to shift a maximum of differential conductance into a minimum is approximately 0.15 mV. The observed F–P resonances reflect the energy spacing Δ*E* between standing wave modes in the cavity. We observe different energy spacings in the whole *V*_bg_ range measured from the observed checkerboard pattern, ranging from Δ*E* = 0.1 – 0.4 meV (Figure 3c and e). This suggests that there is possibly more than one cavity formed in our device. Using energy spacing and cavity length relation,^35^

$$L_{c}=\frac{\hbar^{2}\pi^{2}}{2m\times \Delta E}$$

we estimate a cavity length, *L*_c_ of approximately 300–850 nm. F–P interference is observed when the phase coherence length exceeds the length of the hole scattering centers (mean free path) forming the F–P cavity. Hence, the F–P cavity length provides a direct estimate of the mean free path in the system and a lower bound on the phase coherence length. It is likely that multiple cavities are present, formed by several scattering centers acting as F–P resonators. From the estimated range of cavity lengths, the smaller F–P cavity can be associated with the mean free path (i.e., 300 nm). The phase coherence length scale extracted in our devices is consistent with prior reports on phase coherence length in similar Te systems from weak antilocalization measurments.^38^ To observe ballistic transport, device dimensions smaller than this characteristic length would be required.

Devices measured at a low temperature in thicker Te flakes also exhibit F–P interference. However, we note that visibility (amplitude of conductance w.r.t background) of F–P in thicker flakes is significantly weaker compared to thinner flakes consistent with the picture that thinner flakes provide stronger confinement as compared to thicker flakes (Supplementary Figure S4).

### Magnetic Field Evolution

We now turn to the out-of-plane magnetic field (*B*) dependence in our device. Figure 4a displays a 2D color plot of the evolution of *G* as a function of *V*_bg_ and *B*. The data shown in Figure 4a are plotted after a polynomial background conductance subtraction to improve the *G* peak evolution visibility. The unprocessed measured data are shown in Supplementary Figure S5.

**Figure 4:**
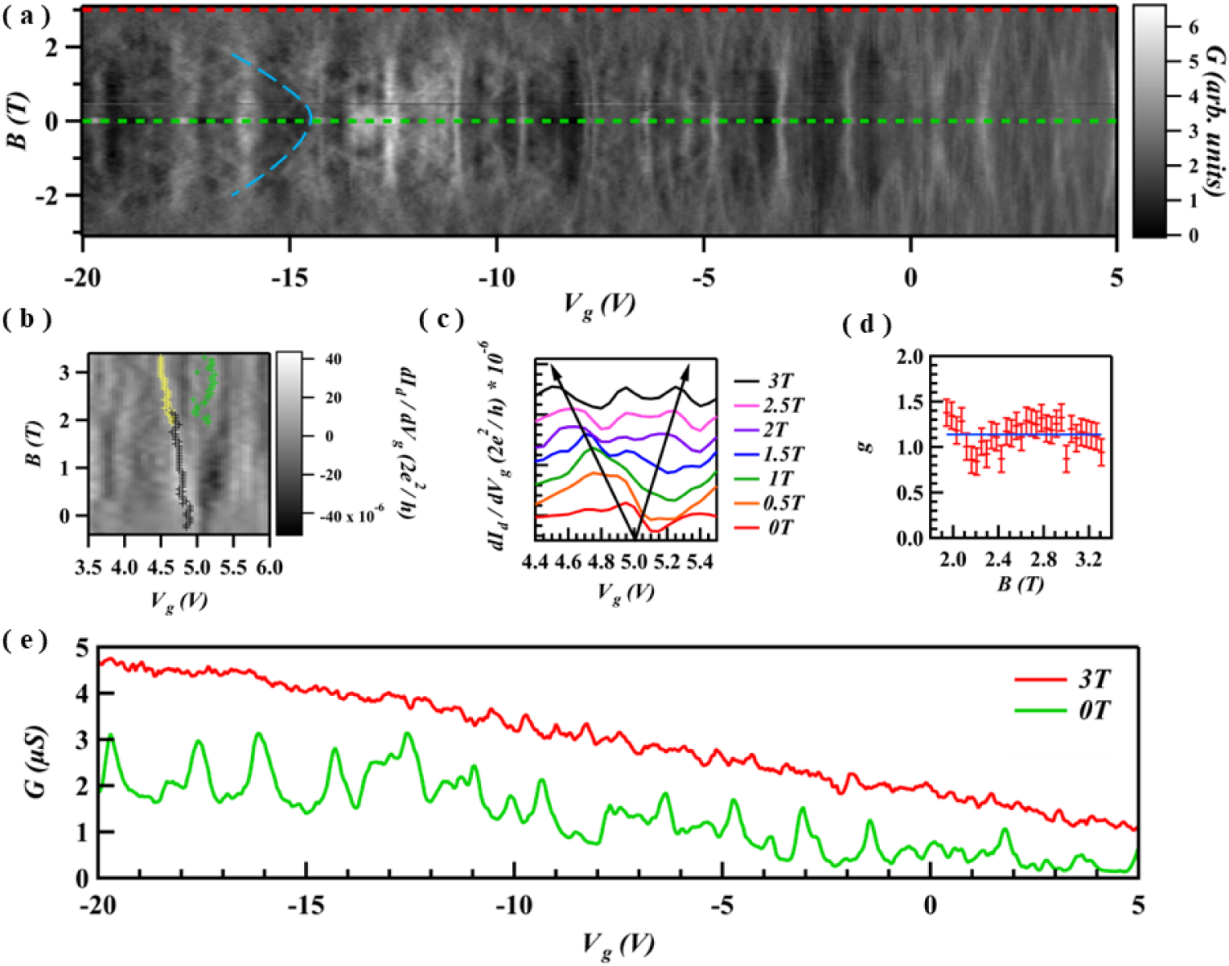
Magneto-transport revealing distinct behaviors for Coulomb blockade and Fabry-Pérot interference. (a) Differential conductance plotted against backgate voltage and magnetic field. Two distinct evolutions are visible: linear dispersion of the Coulomb peaks at low carrier density and a nonlinear dispersion of the Fabry-Pérot interference fringes at higher density, which bend significantly with the field (indicated by the blue dashed line). (b) An example of Zeeman splitting for a single Coulomb peak at a fixed backgate voltage of *V*_bg_ = 5 V. The splitting of the conductance peak is tracked using a software peak-finder. (c) Conductance line cuts taken from panel b at specific magnetic fields displaying peak evolution. (d) The effective g-factor as a function of magnetic field, extracted from the Zeeman splitting shown in (b). (e) Line cuts of conductance traces taken at B = 0 T and B = 3 T. The prominent Fabry-Pérot oscillations observed at zero field completely disappeared at 3 T. This suppression occurs when the cyclotron diameter (2Rc) becomes smaller than the Fabry-Pérot cavity length, effectively preventing the backscattering required for interference.

The observed *G* peak evolves in two ways. One set of *G* peaks, particularly in the lower gate voltage range, exhibits a linear dispersion with *B*-field while some peaks exhibit bending of the oscillation and disappear above *B* = 2 T (Figure 4a). *G* peaks that present a linear dispersion with *B* field is a signature of charge localization, and the linear shift corresponds to the energy level shift due to Zeeman splitting (Figure 4b and c).^46^ From the evolution of these energy levels with a magnetic field (using *E* = *g*μ_B_ *B*), we estimate a *g*-factor of 1.14 (Figure 4d). The experimentally determined g-factor in our device is smaller relative to the free-electron value (*g* ≈ 2). A plausible explanation for this is the renormalization of the Te hole *g*-factor due to its hybridization with the superconducting leads (SnTe).^47^ This suggests that the measured *g*-factor may describe the quasiparticle coherence peaks arising from the superconductor-semiconductor interface. Other factors contributing to the smaller observed g-factor may arise from the strongly anisotropic crystal structure of Te. Moreover, as a heavy element, Te exhibits pronounced spin–orbit coupling, which, together with its anisotropic crystal symmetry, is expected to result in an anisotropic g-factor. To conclusively determine the origin of this behavior, systematic studies of the angular dependence of the g-factor under varying magnetic field orientations are required. A more detailed investigation, potentially employing metallic contacts that remain nonsuperconducting at low temperatures, would further clarify the underlying physical mechanisms.

The F–P conductance oscillations, observed at large negative gate voltages, exhibit a nonlinear dispersion with increasing magnetic field and are fully suppressed above *B* ≈ 2 T (Figure 4e). This behavior is attributed to the Lorentz force bending the hole trajectories within the cavity. The interference vanishes when the cyclotron diameter (2R_c_) becomes smaller than *L*_*c*_, which prevents backscattering and hence multiple reflections forming a standing wave.^46^ At *V*_bg_ = −15 V, we estimate a Fermi velocity of *v*_*f*_ ≈ 6 × 10^5^ m/s. This yields a cyclotron diameter of 2*R*_*c*_ ≈ 336 nm at the 2 T field, a value consistent with the cavity length of *L*_*c*_ ≈ 300–850 nm estimated from the zero-field interference pattern (Figure 3c,e) with the upper limit of the *B*-field value determined by the lower bound of the *L*_*c*_. The red dashed line represents the magnetic field at which the classical cyclotron radius of the charge carriers exceeds the *L*_*c*_.

Here, we address the possibility that the observed conductance modulations arise from universal conductance fluctuations (UCF) rather than F–P interference. Distinguishing between these two mechanisms is essential for validating the interpretation of our data. In principle, UCF can coexist with F–P interference in mesoscopic systems; however, the two phenomena have fundamentally different physical origins and experimental signatures. F–P interference originates from (quasi)­ballistic transport through a well-defined resonant cavity formed between partially reflecting contacts or scattering centers, leading to phase-coherent multiple reflections. In contrast, UCF arises from diffusive transport and quantum interference among many randomly distributed scattering paths in a disordered conductor. Crucially, UCF is characterized by aperiodic yet reproducible conductance fluctuations with a typical amplitude on the order of *e* ^2^/h and does not give rise to stable, periodic patterns in bias–gate-voltage space.

By comparison, our Te flakes exhibit relatively regular, periodic checkerboard patterns in two-dimensional bias–gate-voltage maps, a clear π-phase shift between constructive and destructive interference (Figure 3g), and a systematic evolution with a magnetic field that is quantitatively consistent with cyclotron-radius considerations and a cavity length extracted from the checkerboard pattern (Figure 4a). The oscillation periods obtained from the data directly correlate with the expected F–P cavity dimensions (much smaller than the device dimensions), further supporting a quasi-ballistic interference origin. While a weak, aperiodic UCF background cannot be entirely excluded, it cannot account for the pronounced periodicity, phase coherence, and magnetic-field dependence observed here. We therefore conclude that F–P interference is the dominant transport mechanism in our Te devices, with any contribution from UCF playing at most a minor, secondary role.

## Conclusions

In summary, we fabricated and characterized field-effect devices based on thin tellurium flakes, demonstrating their potential as high-quality platforms for quantum transport studies. We demonstrate a high carrier mobility in 17 nm thick flake reaching a value of 1000 cm^2^/V·s at 30 K. At low temperatures, we observed a clear evolution from Coulomb blockade to Fabry-Pérot interference, with the visibility of these quantum oscillations being notably enhanced in thinner flakes. The application of a magnetic field allowed for the direct observation of the Zeeman splitting.

The combination of high mobility and gate-tunable quantum phenomena piques interest for future explorations in thin tellurium flakes. The measurements also shed light on the device dimensions necessary to access the ballistic regime where individual sub-band transport could be explored. We expect a further enhancement in carrier mobility and mean free path by encapsulating the flake in boron nitride. The results presented here pave the way for more complex device geometries and experiments aimed at harnessing the unique chiral and topological properties of tellurium. This includes investigating predicted Weyl physics, realizing topological superconductivity in hybrid devices, and developing low-power spintronic components.

## Methods

### Sample Preparation

Synthetic method of 2D Te nanosheets: The synthesis of tellurene nanosheets was achieved through a sequential process utilizing metastable 1T’-MoTe_2_ as dual-functional templates and Te sources in *N*-methylpyrrolidone (NMP) solvent. Initially, 1T’-MoTe_2_ crystals (3 mg/mL) underwent prolonged bath ultrasonication (140 W, 10 h), followed by a 5 h quiescent incubation period. The resultant gray translucent suspension was then homogenized through brief low-power sonication (60 W, 1 min) before undergoing a purification sequence involving primary centrifugation (3,000 rpm, 1 min), ethanol-assisted flocculation, and two dispersion-centrifugation cycles to yield stable tellurene colloids. For device fabrication, controlled deposition was executed by dropping the purified suspension onto SiO_2_/Si substrates followed by instantaneous nitrogen-flow drying to isolate individual nanosheets.

### Device Fabrication

Device fabrication was carried out using a maskless photolithography writer to pattern the source and drain contacts in a two-step process. For the initial step, a bilayer photoresist was spin-coated onto the sample, and fine contacts designed to overlap the flake were defined. These contacts were then metallized by depositing Sn/Au or Pd/Au (20 nm/60 nm), followed by a standard lift-off procedure. Subsequently, the sample was coated again with the bilayer photoresist to pattern large-area contact pads connected to fine leads. These pads were metallized with Cr/Au (5/80 nm) to ensure robust connections for wire bonding.

## Supplementary Material



## Notes

S1. AFM measurement in thick flakes. S2. Conductance on–off ratio in thick flake device. S3. Temperature-dependent mobility in thick flake (20 nm). S4. Fabry–Perot interference in thick Te flake devices. S5. As-measured magnetic field evolution of Figure 4a in the main text (PDF)
