# Supplementary material for: Phase-Coherent Transport in Two-Dimensional Tellurium Flakes
Source: ACS Appl Electron Mater. 2026 Feb 4;8(4):1539–46. doi: 10.1021/acsaelm.5c01853 (PMC12937097; doi:10.1021/acsaelm.5c01853)
Supplement: Supplementary file 1 [file el5c01853_si_001.pdf]

Supporting Information for "Phase Coherent Transport in Two-Dimensional Tellurium Flakes"

*Mohammad Hafijur Rahaman1, Nathan Tanner Sawyers1, Mourad Benamara2, Trudie Culverhouse3,4, Gokul Acharya1, Durga Venkata Maheswar Repaka3, Qiyuan He5, Hugh O. H. Churchill1,6, Dharmraj Kotekar Patil1,6**

[1]Department of Physics, University of Arkansas, Fayetteville, AR 72701 USA

[2]Institute for Nano Science and Engineering, University of Arkansas, Fayetteville, AR 72701 USA

[3] Institute of Materials Research and Engineering, Agency for Science Technology and Research, (A*STAR) Singapore 138634, Republic of Singapore 138634

[4]Department of Chemistry, School of Natural Sciences, University of Manchester, Manchester M13 9PL,United Kingdom

[5]Department of Materials Science and Engineering, City University of Hong Kong, 83 Tat Chee Avenue, Kowloon, Hong Kong, China

[6]MonArk NSF Quantum Foundry, University of Arkansas, Fayetteville, AR 72701 USA

*corresponding email: dk030@uark.edu

Figure S1. AFM measurement in thick flakes
Figure S2. Conductance on-off ratio in thick flake device
Figure S3. Temperature dependent mobility in thick flake (20 nm)
Figure S4. Fabry-Perot interference in thick Te flake devices
Figure S5. As measured magnetic field evolution of Figure 4a in the main text

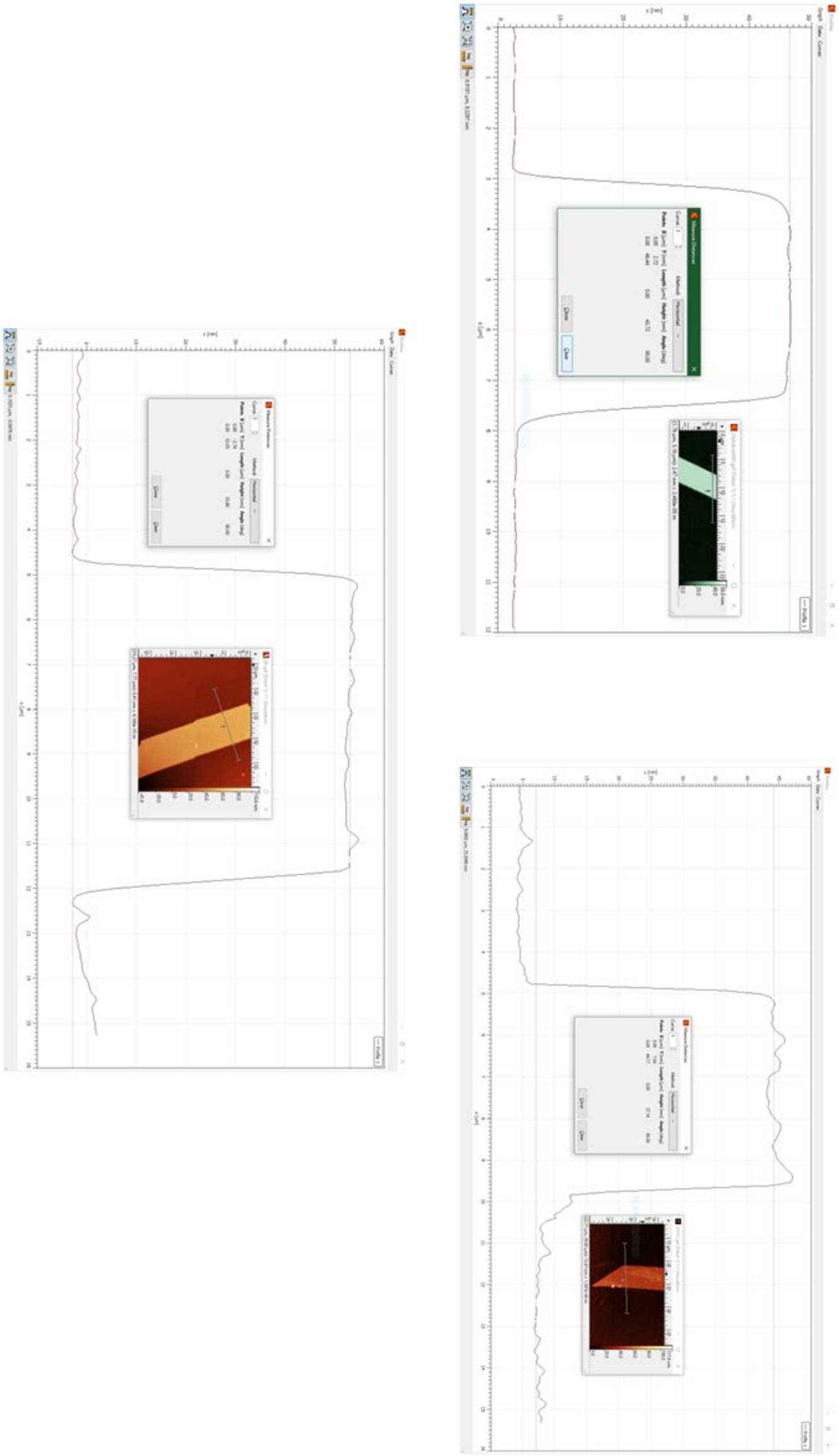

Figure S1: Atomic Force Microscope images of thick Te flakes.

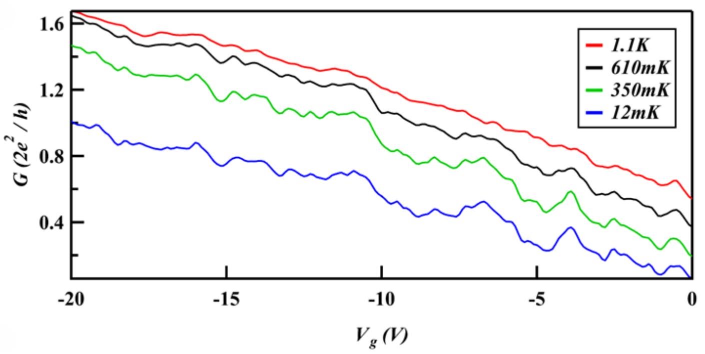

Figure S2: Line trace G vs V_{bg} exhibiting weak gate dependence with on-off ratio of <10 in flake with thickness > 40nm.

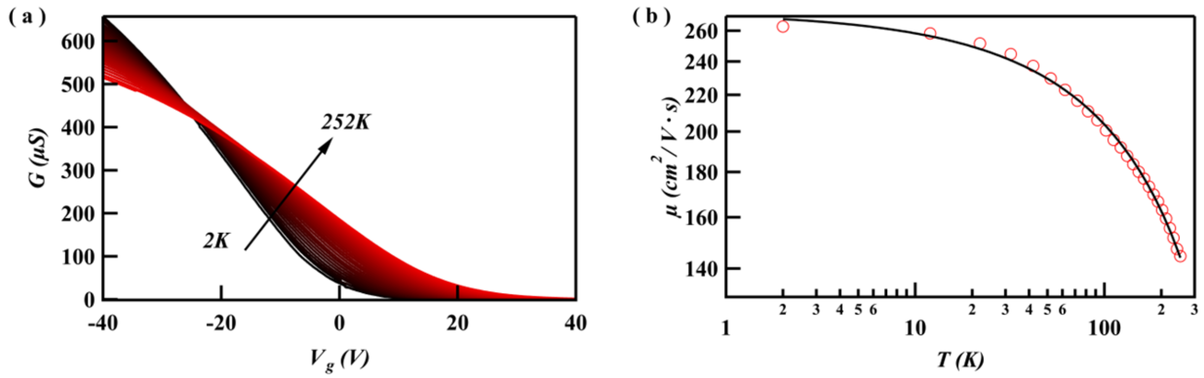

Figure S3: Conductance as a function of backgate and temperature in a Te device with thickness 20nm. Lower mobility is observed in this device compared to thinner flake device shown in the main text.

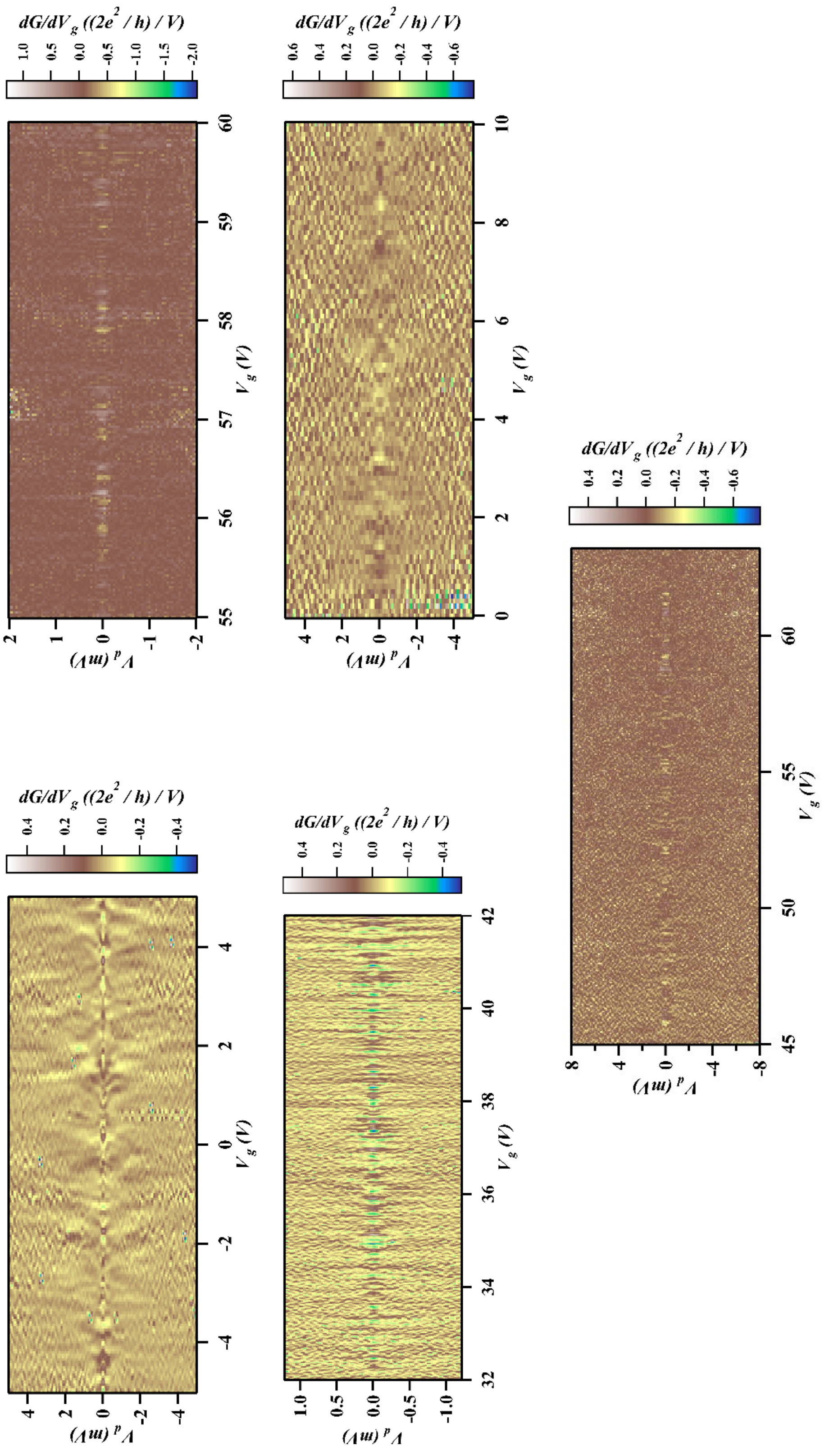

Figure S4: Low temperature characterization of thick Te flakes (thickness of 40 nm and above) exhibiting F-P interference. The observed F-P interference is significantly weak in thicker flakes as compared to thin flake shown in the main text.

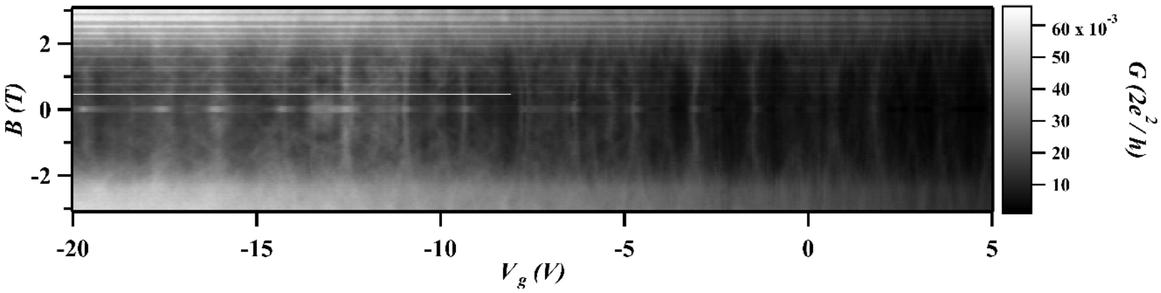

Figure S5: As measured conductance as a function of backgate and magnetic field for the data shown in figure 4a of the main text. The switching behavior and background is corrected for to display clear evolution of conductance peaks.
